# Hepatic Artery Pseudoaneurysm in an Infant With Pancreatitis

**DOI:** 10.7759/cureus.59348

**Published:** 2024-04-30

**Authors:** Nawal M Alrubia, Nouryah A Alhafez

**Affiliations:** 1 Pediatric Gastroenterology, Maternity and Children's Hospital, Dammam, SAU; 2 Medicine, Alfarabi College, Riyadh, SAU

**Keywords:** case report, infants, pancreatitis, hepatic, pseudoaneurysm

## Abstract

Hepatic artery pseudoaneurysm (HAP) is an uncommon yet critical complication of acute pancreatitis. This case delves into the unusual scenario of a two-month-old male infant with a familial history of pancreatitis who develops the condition himself. Despite initial treatment, the infant's symptoms worsened, unveiling a pancreatic pseudocyst (PCC) and an atypical pseudoaneurysm stemming from the hepatic artery, a rare complication in acute pancreatitis. The pseudoaneurysm's confirmation through selective angiography and its subsequent management using embolization is highlighted.

This report emphasizes the rarity of hepatic artery pseudoaneurysm in the context of acute pancreatitis, stressing the need for thorough imaging to spot arterial involvement. Early identification via selective angiography remains crucial due to the high risks associated with pseudoaneurysm rupture, underscoring the urgency for prompt intervention.

In summary, this case spotlights the infrequent occurrence of hepatic artery pseudoaneurysm secondary to acute pancreatitis in an infant. It stresses the importance of swift recognition and intervention to avert potentially life-threatening complications.

## Introduction

Visceral artery pseudoaneurysm represents a rare yet perilous complication arising from chronic pancreatitis (CP). Predominantly, the splenic artery exhibits the highest incidence, succeeded by the gastroduodenal artery, with the involvement of the hepatic artery being less frequent in occurrence [1]. Acute pancreatitis can progress severely in 20%-30% of cases, leading to life-threatening complications such as multi-organ failure and hemorrhage due to the development of pseudoaneurysms or pseudocysts [2]. Pseudoaneurysms account for 4%-10% of these complications [3]. The mortality rate can reach up to 90% if a pseudoaneurysm rupture is left untreated. Nonetheless, with appropriate treatment, the mortality rate can be reduced to between 15% and 50% [2]. As a result, prompt diagnosis and effective medical management are critical. Transcatheter arterial embolization is acknowledged as a safe and effective intervention for this type of pseudoaneurysm [4]. A hepatic artery pseudoaneurysm (HAP) is an uncommon condition stemming from acute or chronic surgical trauma to the hepatic artery. Sethi et al. [5] reported that pancreatic pseudoaneurysms occur in the hepatic artery in 19% of cases. These pseudoaneurysms usually occur in the common or proper hepatic arteries, not the peripheral arterial branches. This leads to a higher probability of incidence in the extrahepatic region [6]. Although there is existing literature about the prevalence and treatment of pseudoaneurysms in chronic pancreatitis, there is limited documentation regarding their occurrence in acute pancreatitis [2,7]. The clinical manifestation and therapeutic modalities for hepatic artery pseudoaneurysm are not well documented. Furthermore, there are no literature reviews on this rare complication. Therefore, we present a case report.

## Case presentation

**Figure 1:**
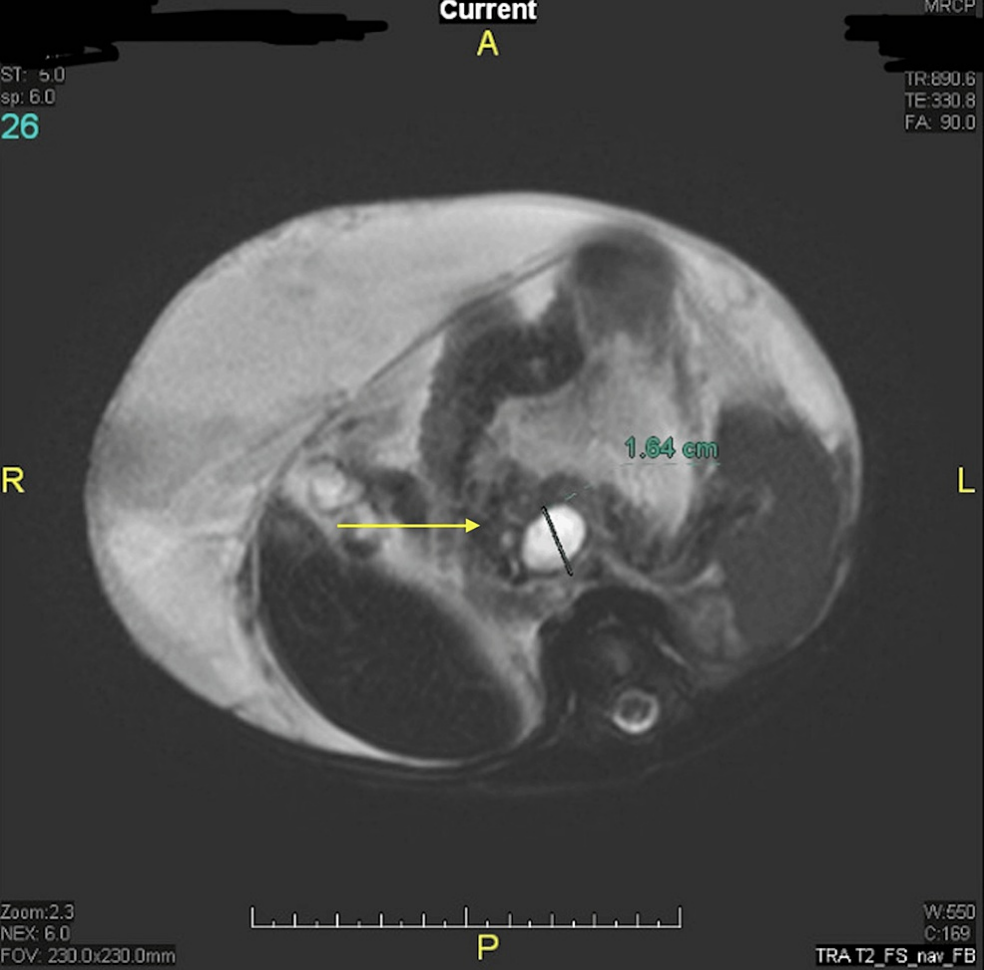
MRCP demonstrating a large filling defect arising from the main pancreatic duct at the junction of the head and body of the pancreas (arrow) MRCP: magnetic resonance cholangiopancreatography

A two-month-old Saudi infant presents with a 10-day history of vomiting, diarrhea, and abdominal distension. Notably, the infant's grandmother had a history of recurrent pancreatitis requiring enzyme replacement therapy. On examination, a thin male with weight and length measurements falling below the third percentile presented with ascites as the sole abnormal clinical manifestation. Initial evaluations encompassing routine hematological, hepatic, and renal profiles alongside metabolic assessments, including sweat chloride test, triglyceride, and cholesterol levels, displayed normal results. Ascitic tap analysis revealed hemorrhagic fluid with unremarkable cytological and bacteriological examinations yet notably elevated protein content (46 g/L), amylase (17,300 U/dL), and lipase (20,000 U/dL). Serum amylase levels were measured at 1,600 U/dL, while serum lipase levels registered at 2,300 U/dL. Genetic analysis targeting the* PRSS1* gene mutation exhibited normal findings, effectively excluding hereditary pancreatitis. Imaging modalities such as ultrasound and computed tomography (CT) of the abdomen identified only the presence of ascites, while magnetic resonance cholangiopancreatography (MRCP) illustrated a substantial filling defect originating from the main pancreatic duct at the junction of the pancreas's head and body (Figure 1).

**Figure 2:**
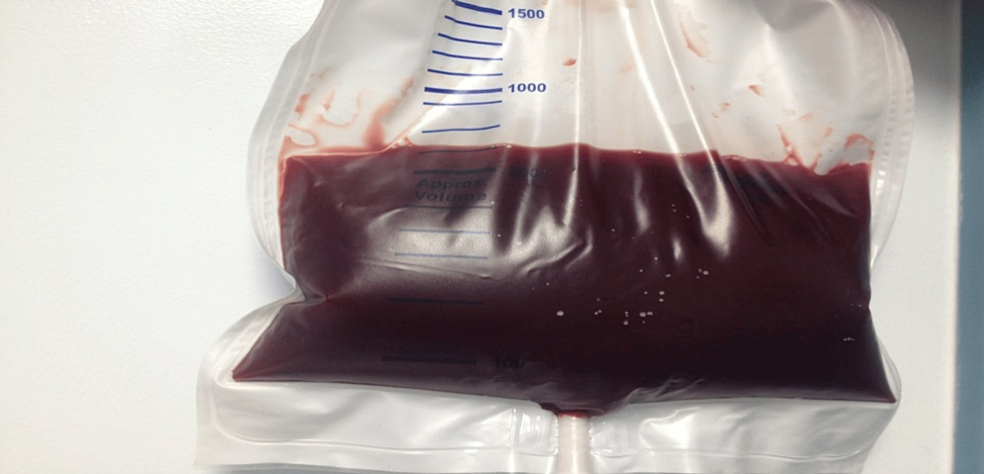
Ascitic tap (>2 liters of blood-stained fluid was drained)

The diagnosis confirmed acute pancreatitis. Upon readmission, the patient presented with escalating abdominal distension, weakness, and pallor, accompanied by a hemoglobin level of 6 g% (normal: 10 g%). Ascitic tap revealed the presence of bloody fluid (Figure 2).

**Figure 3:**
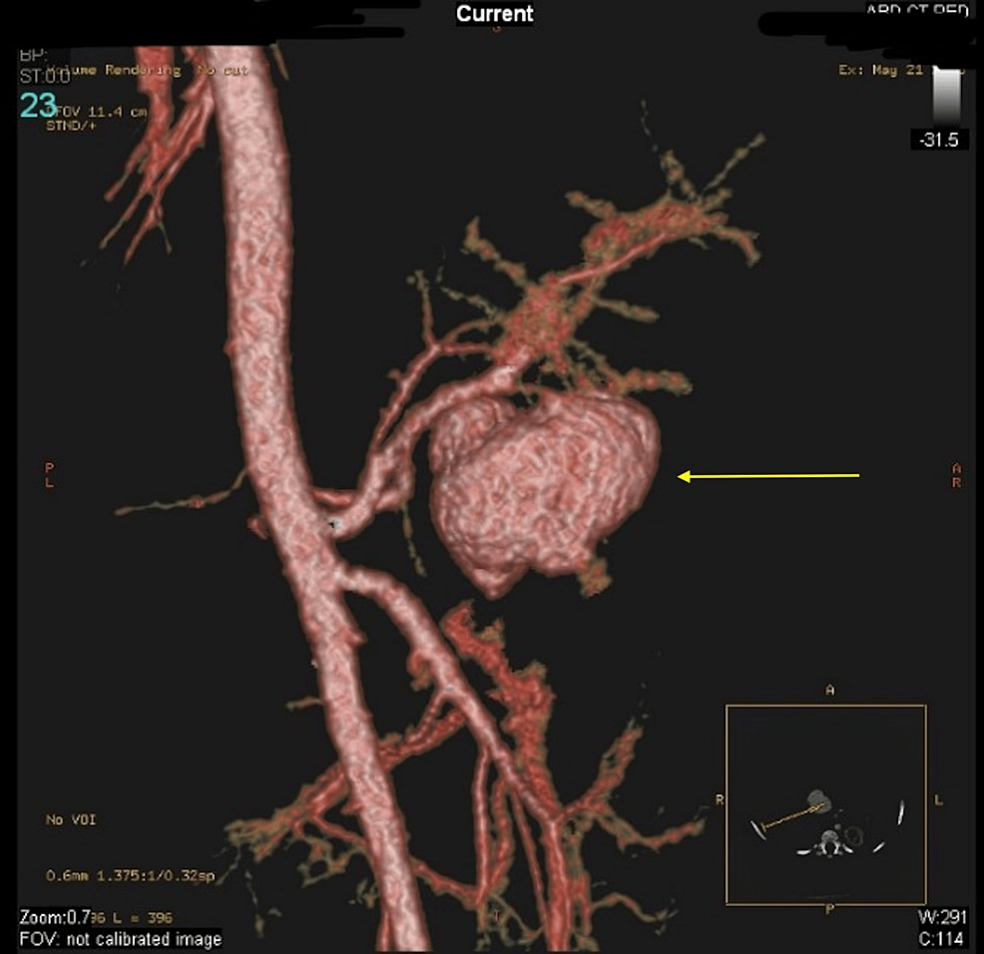
3D CT images confirming the communication of the pseudoaneurysm from the hepatic artery with a turbulent flow at this level (arrow) 3D CT: three-dimensional computed tomography

Ascites reappeared two months later, prompting a paracentesis procedure involving continuous drainage for three weeks, yielding over 2 liters of blood-stained fluid. Subsequent ultrasounds revealed a pancreatic pseudocyst (PCC) with irregular blood flow. Further 3D CT angiography images confirmed the communication of the pseudoaneurysm from the hepatic artery, exhibiting turbulent flow at this juncture (Figure 3).

The patient was scheduled for selective angiography and embolization. However, on the day of the procedure, a transabdominal ultrasound with Doppler was performed and revealed an absence of blood flow, indicating a potential thrombus formation. As a result, the embolization procedure was canceled.

**Figure 4:**
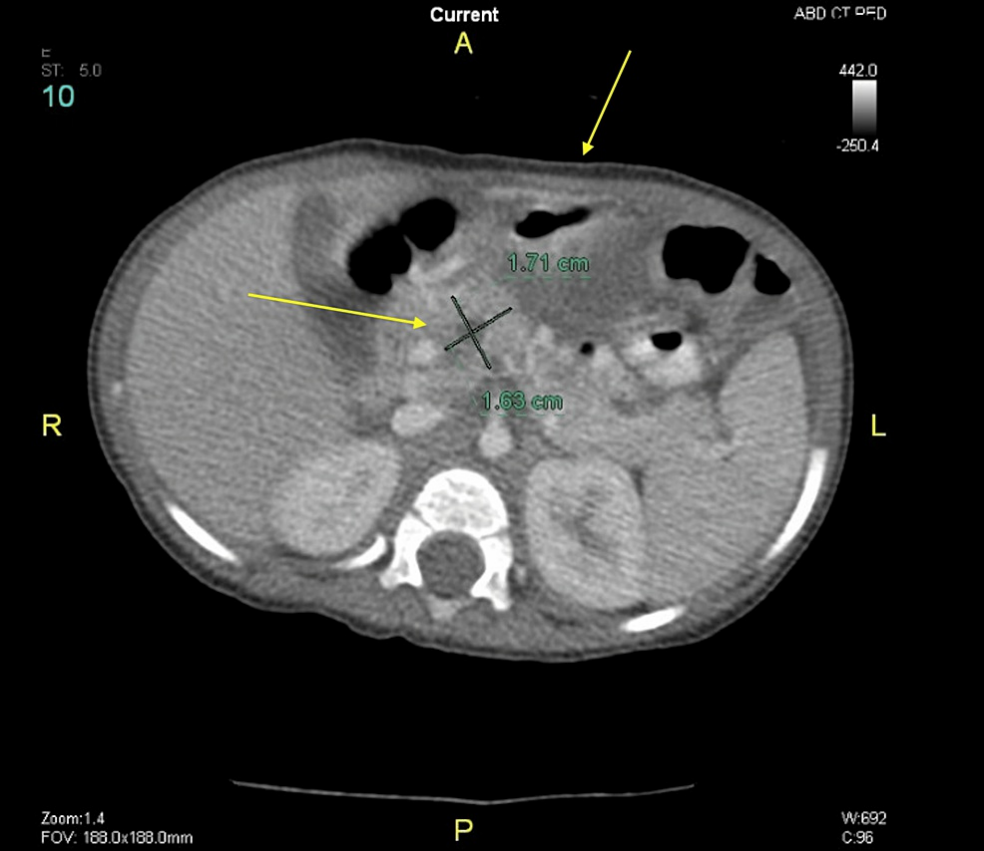
Abdominal CT scan showing pseudocyst in the head of the pancreas (arrows) CT: computed tomography

**Figure 5:**
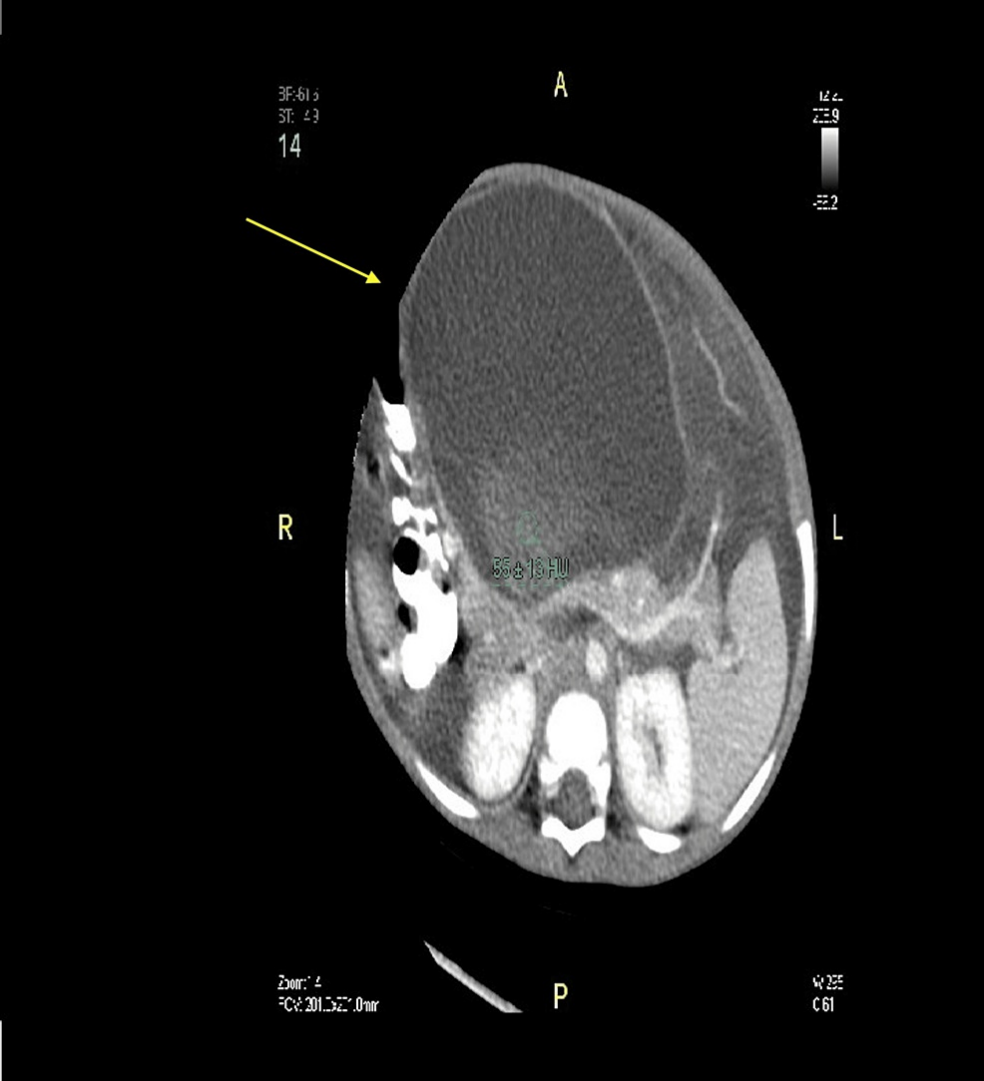
Abdominal CT scan showing pseudocyst in the head of the pancreas surrounded by fluid after follow-up (arrow) CT: computed tomography

**Figure 6:**
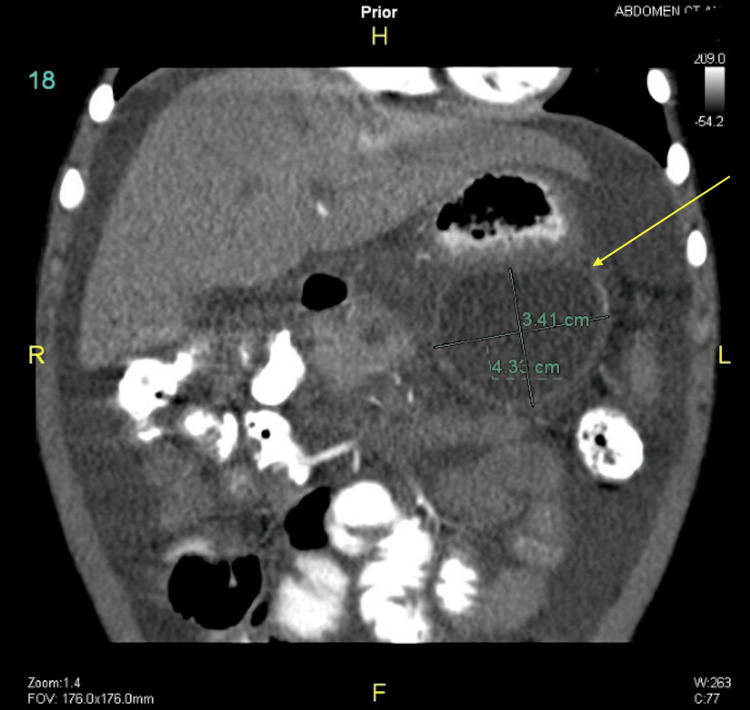
CT scan showing pseudocyst in the body and tail of the pancreas (arrow) CT: computed tomography

**Figure 7:**
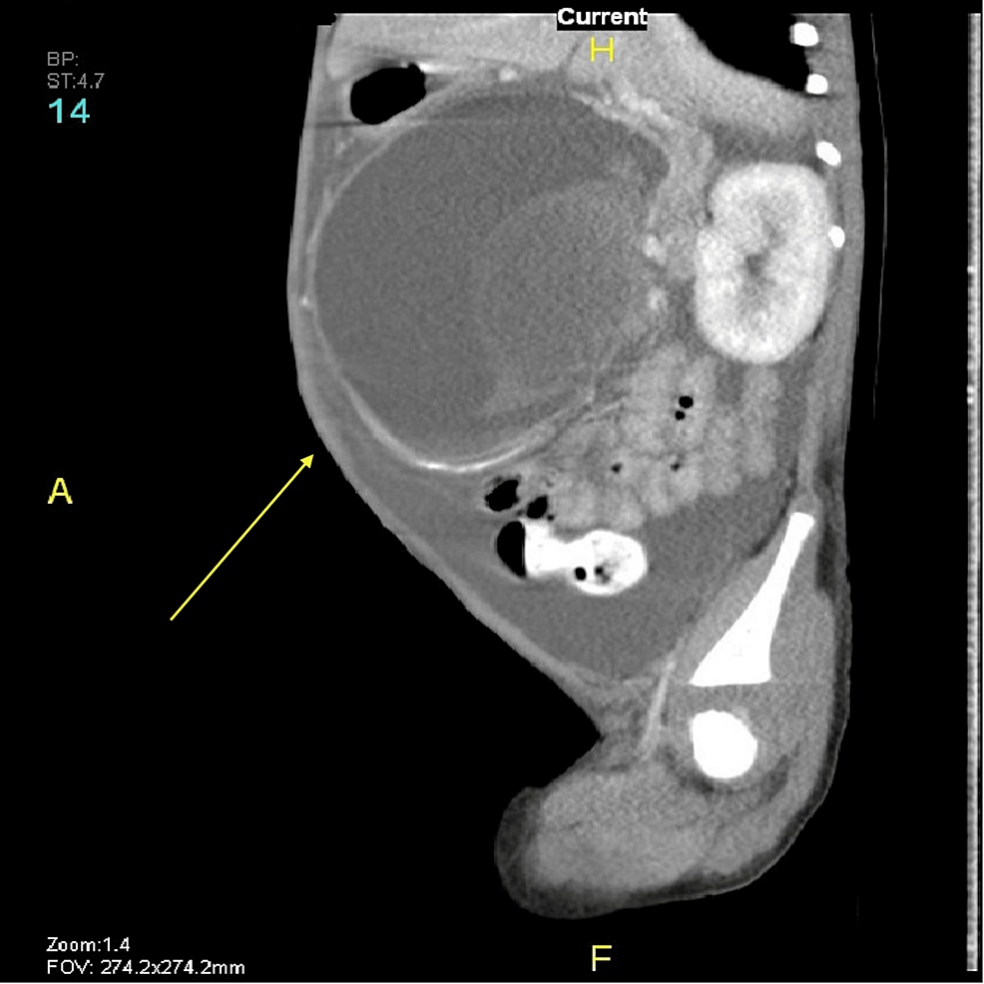
Abdominal CT scan showing pseudocyst in body and tail of the pancreas surrounded by fluid after follow-up (arrow) CT: computed tomography

Following regular outpatient clinic visits, routine laboratory tests, and abdominal CT scans, the patient was found to have two large pseudocysts. Initially, one was discovered in the head of the pancreas, and three months later, another was found in the body and tail of the pancreas (Figures 4-7).

The patient underwent an exploratory laparotomy where both pseudocysts were drained. The pseudocysts were found to contain fluid and granulation tissue. A cystogastrostomy was performed during the operation, which lasted three hours without any blood loss. Biopsies of the peritoneum and omentum showed inflammatory granulation tissue. The patient's postoperative hospital stay was one week, and his course was uneventful. Twelve months later, a CT scan of the abdomen showed a normal pancreas without cysts. He has gained 8 kg in weight and remains free of ascites.

## Discussion

We present a rare occurrence of hepatic artery pseudoaneurysm associated with acute pancreatitis, accompanied by pre- and post-development imaging findings. Previous studies, such as the work by Sethi et al. [5], reported that pseudoaneurysms resulting from pancreatitis primarily manifest in the splenic artery (40%), gastroduodenal artery (30%), pancreaticoduodenal artery (20%), gastric artery (5%), and hepatic artery (2%). This underscores their frequent occurrence in proximal arteries prone to fluid retention, particularly among arteries closely linked to the pancreas, hinting at the potential for pseudoaneurysm formation in the common or proper hepatic arteries [6,8].

While isolated cases of hepatic artery pseudoaneurysms complicating pancreatitis have been documented [7], only two instances of right hepatic artery pseudoaneurysm distal to the proper hepatic artery have been reported [9,10]. Notably, these two cases involved anomalous hepatic artery divisions. However, in our case, the inflammation appeared to have diffused across a broader area, and the enzyme-rich fluid that dispersed around the pancreas likely infiltrated along Glisson's sheath, contributing to the formation of a right hepatic artery pseudoaneurysm. Consequently, the potential for pseudoaneurysm development exists in any area susceptible to fluid infiltration. Given the life-threatening risk associated with pseudoaneurysm rupture, meticulous assessment of both pancreatic-associated arteries and those within a broader scope, including distal sections of the hepatic artery, is imperative during initial imaging and subsequent follow-ups. Hepatic artery pseudoaneurysm rupture occurs frequently, accounting for up to 76% of cases [3], emphasizing the criticality of early detection and intervention. While intrahepatic artery pseudoaneurysms constitute approximately 20% of all hepatic artery pseudoaneurysms, most stem from complications arising from percutaneous procedures such as transhepatic cholangiography, transhepatic drainage catheter placement, or liver biopsy [11]. Therefore, recognizing acute pancreatitis as a rare cause of intrahepatic pseudoaneurysm is crucial.

An analysis of seven cases of hepatic artery pseudoaneurysm by Finley et al. [8] revealed four instances developing in the common hepatic artery in the extrahepatic region, one in the right hepatic artery in the extrahepatic region, and two in the intrahepatic branch of the right hepatic artery. A recent report by Shiozawa et al. [2] also documented a pseudoaneurysm involving the right hepatic artery as a complication of acute pancreatitis.

Our case of hepatic artery pseudoaneurysm was a result of acute pancreatitis, but the communication of the pseudoaneurysm from the hepatic artery with a turbulent flow is also unusual. The most sensitive test for detecting hepatic artery pseudoaneurysm is selective angiography, which was followed in this case [7]. A pseudoaneurysm usually develops 3-5 weeks after the onset of acute pancreatitis, but pseudoaneurysm hemorrhage may occur from a few days to several years after the onset of pancreatitis [3,5]. In cases of an arterial pseudoaneurysm associated with pancreatitis, the primary approach for management involves considering coil embolization as the initial intervention, particularly if the patient's hemodynamic status remains stable [6]. Following coil embolization, this patient underwent laboratory investigations and an abdominal CT scan, revealing a pseudocyst in the pancreatic head. Three months later, another pseudocyst was detected, this time located in the body and tail of the pancreas. Pancreatic pseudocyst (PCC) is an exceptionally uncommon medical condition among children [12]. Sixty percent resulted from trauma, while the cause remained unknown in 32% of cases [13]. The preferred standard method to drain pseudocysts situated in the body and tail of the pancreas is through cystogastrostomy [14]. The same procedure was done here and went uneventfully. The patient on follow-up started to gain weight in the 12th month and remained ascites-free. The identical procedure was performed without any complications. Endovascular stenting is a viable management option for arterial pseudoaneurysms associated with pancreatitis. It involves placing a stent in the affected artery to support its structure and prevent rupture. Being a minimally invasive procedure, it may be favored due to its lower risk of complications compared to open surgery. However, embolization is considered the treatment of choice in such cases [7].

## Conclusions

This case report sheds light on a rare yet critical complication: hepatic artery pseudoaneurysm arising from acute pancreatitis. Detecting such pseudoaneurysms, even in distal hepatic artery sections, is vital due to their potential for life-threatening rupture. Successful management through selective angiography and embolization resulted in subsequent follow-ups revealing pseudocysts, managed without complications via cystogastrostomy. This emphasizes the need for early detection, intervention, and continuous monitoring to avert severe complications, underscoring the importance of further studies to refine therapeutic strategies for this perilous complication.

## References

[1] Dhali A, Ray S, Sarkar A (2022). Peripancreatic arterial pseudoaneurysm in the background of chronic pancreatitis: clinical profile, management, and outcome. Updates Surg.

[2] Shiozawa K, Watanabe M, Ikehara T (2013). Right hepatic artery pseudoaneurysm complicating acute pancreatitis: a case report. Med Princ Pract.

[3] Bergert H, Hinterseher I, Kersting S, Leonhardt J, Bloomenthal A, Saeger HD (2005). Management and outcome of hemorrhage due to arterial pseudoaneurysms in pancreatitis. Surgery.

[4] Balthazar EJ, Fisher LA (2001). Hemorrhagic complications of pancreatitis: radiologic evaluation with emphasis on CT imaging. Pancreatology.

[5] Sethi H, Peddu P, Prachalias A, Kane P, Karani J, Rela M, Heaton N (2010). Selective embolization for bleeding visceral artery pseudoaneurysms in patients with pancreatitis. Hepatobiliary Pancreat Dis Int.

[6] Siegelman SS, Copeland BE, Saba GP, Cameron JL, Sanders RC, Zerhouni EA (1980). CT of fluid collections associated with pancreatitis. AJR Am J Roentgenol.

[7] Singh CS, Giri K, Gupta R, Aladdin M, Sawhney H (2006). Successful management of hepatic artery pseudoaneurysm complicating chronic pancreatitis by stenting. World J Gastroenterol.

[8] Finley DS, Hinojosa MW, Paya M, Imagawa DK (2005). Hepatic artery pseudoaneurysm: a report of seven cases and a review of the literature. Surg Today.

[9] Rao RC, Kumar A, Berry M (1987). Pseudoaneurysm of anomalous right hepatic artery as a cause for hemosuccus pancreatitis. Gastrointest Radiol.

[10] Falkoff GE, Taylor KJ, Morse S (1986). Hepatic artery pseudoaneurysm: diagnosis with real-time and pulsed Doppler US. Radiology.

[11] Rösch J, Petersen BD, Hall LD, Ivancev K (1990). Interventional treatment of hepatic arterial and venous pathology: a commentary. Cardiovasc Intervent Radiol.

[12] Pareek P, Agrawal N (2016). Pancreatic pseudocyst in children: a rare medical entity. Int Surg J.

[13] Cooney DR, Crosfeld JL (1975). Operative management of pancreatic pseudocysts in infants and children: a review of 75 cases. Ann Surg.

[14] Khalifa M, Gobran T, Shreef KS, Waly A (2015). Pancreatic pseudocyst in children: a single‐institute experience. Ann Pediatr Surg.

